# Sodium ion stress accelerates cell lysis in *Bacillus coagulans* by exacerbating energy deficit during carbon source transition

**DOI:** 10.1128/aem.01314-26

**Published:** 2026-08-05

**Authors:** Zhihao Liu, Yingping Zhuang, Yonghong Wang, Guan Wang

**Affiliations:** 1 State Key Laboratory of Photoelectric Conversion and Utilization of Solar Energy, Qingdao Institute of Bioenergy and Bioprocess Technology, Chinese Academy of Sciences 85437, Qingdao, China; 2 Shandong Energy Institute, Qingdao, China; 3 Qingdao New Energy Shandong Laboratory, Qingdao, China; 4 State Key Laboratory of Bioreactor Engineering, East China University of Science and Technology 47860, Shanghai, China; Universita degli Studi di Napoli Federico II, Portici, Italy

**Keywords:** mixed carbon sources, neutralizer, cell lysis, omics analysis, energy supply

## Abstract

The choice of pH neutralizer plays a critical role in determining the performance of industrial lactic acid fermentation. This study investigated the distinct phenotypic and metabolic responses of *Bacillus coagulans* during batch fermentation with mixed carbon sources (glucose and trehalose) when using sodium hydroxide versus calcium hydroxide for pH control. Compared to calcium hydroxide, sodium hydroxide resulted in a slightly lower lactic acid yield and induced significant cell lysis during the carbon source transition. Metal ion experiments confirmed that sodium ion stress is the primary factor responsible for extensive cell lysis. Omics analysis indicated that under sodium hydroxide conditions, cells require more energy to resist stress. To meet this demand, cells downregulated cellular growth and sodium ion uptake systems, while upregulating carbon source transport. However, during carbon source transition, cellular resource reallocation resulted in insufficient carbon source uptake, leading to stagnation of glycolysis and amino acid metabolism. The resulting energy deficit triggered upregulation of cellular hydrolases, ultimately causing population-wide cell lysis. In contrast, calcium hydroxide as a neutralizer does not impose sodium stress and contributes to maintaining cell wall stability under carbon source deficiency. This study contributes to the understanding of cellular regulatory mechanisms in response to different neutralizers, and provides insights for metabolic engineering and process optimization.

**IMPORTANCE:**

pH neutralizer selection critically determines cell survival and process efficiency in industrial fermentation. Our study demonstrates that sodium hydroxide, although effective for pH control, imposes severe sodium ion stress that synergizes with the inherent energy vulnerability of cells during carbon source transition, triggering catastrophic population-wide lysis in *Bacillus coagulans*. Through integrated physiology, ion experiments, and multi-omics analyses, we reveal that the underlying cause is an energy crisis arising from the conflict between stress defense and metabolic reallocation. In contrast, calcium hydroxide not only avoids sodium stress but also confers structural and thermal stability to cells, thereby preserving cell density and improving carbon utilization. These findings shift the perspective on neutralizers from mere pH regulators to key determinants of cellular energy economy and survival. Our study thus offers a rational basis for neutralizer selection, strain engineering toward ion resistance, and the design of robust fermentation processes, with direct implications for the sustainable and cost-effective production of lactic acid and other microbial metabolites.

## INTRODUCTION

Lactic acid, as the key monomer for synthesizing the biodegradable plastic polylactic acid (1), relies on efficient and low-cost microbial fermentation for sustainable production (2–4). During industrial lactate fermentation, neutralizers must be continuously added to maintain the optimal pH range, thereby ensuring sustained microbial growth and product synthesis (5).

However, the choice of neutralizer profoundly influences both the economic viability and environmental footprint of the overall process. Calcium hydroxide [Ca(OH)_2_] or calcium carbonate (CaCO_3_) is commonly used in industrial lactic acid fermentation (6–8); nevertheless, during subsequent lactic acid extraction and purification, sulfuric acid is added to convert calcium lactate into lactic acid, co-producing large amounts of gypsum (CaSO_4_) (5, 9). Consequently, sodium hydroxide (NaOH) or ammonium hydroxide (NH_4_OH) is also employed as alternatives to avoid calcium salt handling (5, 10). Nevertheless, lactate in sodium or ammonium salt forms often exerts stronger inhibitory effects on production strains, leading to reduced final product concentration and lower sugar-to-acid conversion efficiency (11, 12). Current research on neutralizers has largely focused on fermentation process control, while studies on how these agents affect cellular metabolism remain limited. Although some omics-based studies have examined the metabolic impacts of neutralizers (12–14), most were conducted under a single, abundant carbon source condition. In practical industrial biomanufacturing, the use of mixed carbon sources derived from starch hydrolysate has become increasingly common to reduce costs and improve resource efficiency (15, 16). Different carbon sources vary substantially in metabolic pathways, transmembrane transport, and energy demands. When the stress induced by neutralizers overlaps with the dynamic process of carbon source transition, more complex cellular physiological responses may arise. Therefore, the mechanisms underlying the effects of neutralizers on mixed-carbon-source lactic acid fermentation remain poorly understood.

Against this background, this study employs *Bacillus coagulans* (17, 18), an industrially dominant lactic acid producer, to investigate the physiological and metabolic impacts of calcium hydroxide and sodium hydroxide as neutralizers in a mixed carbon source fermentation system containing glucose and trehalose (19). Notably, when sodium hydroxide is used as the neutralizer, it accelerates cell lysis during the carbon source transition phase, whereas calcium hydroxide better maintains cellular integrity. To elucidate this phenomenon, this study combined metal ion experiments, metabolomics, and transcriptomics to analyze the causes of cell lysis and the underlying metabolic response mechanisms. This work not only clarifies the physiological mechanisms of different neutralizers in a dynamic mixed-carbon-source fermentation system but also provides a solid theoretical basis for rational neutralizer selection, optimization of fermentation processes, and metabolic engineering aimed at improving stress resistance in industrial strains.

## RESULTS

### Effect of different neutralizers on lactate fermentation under mixed carbon sources

**Fig 1:**
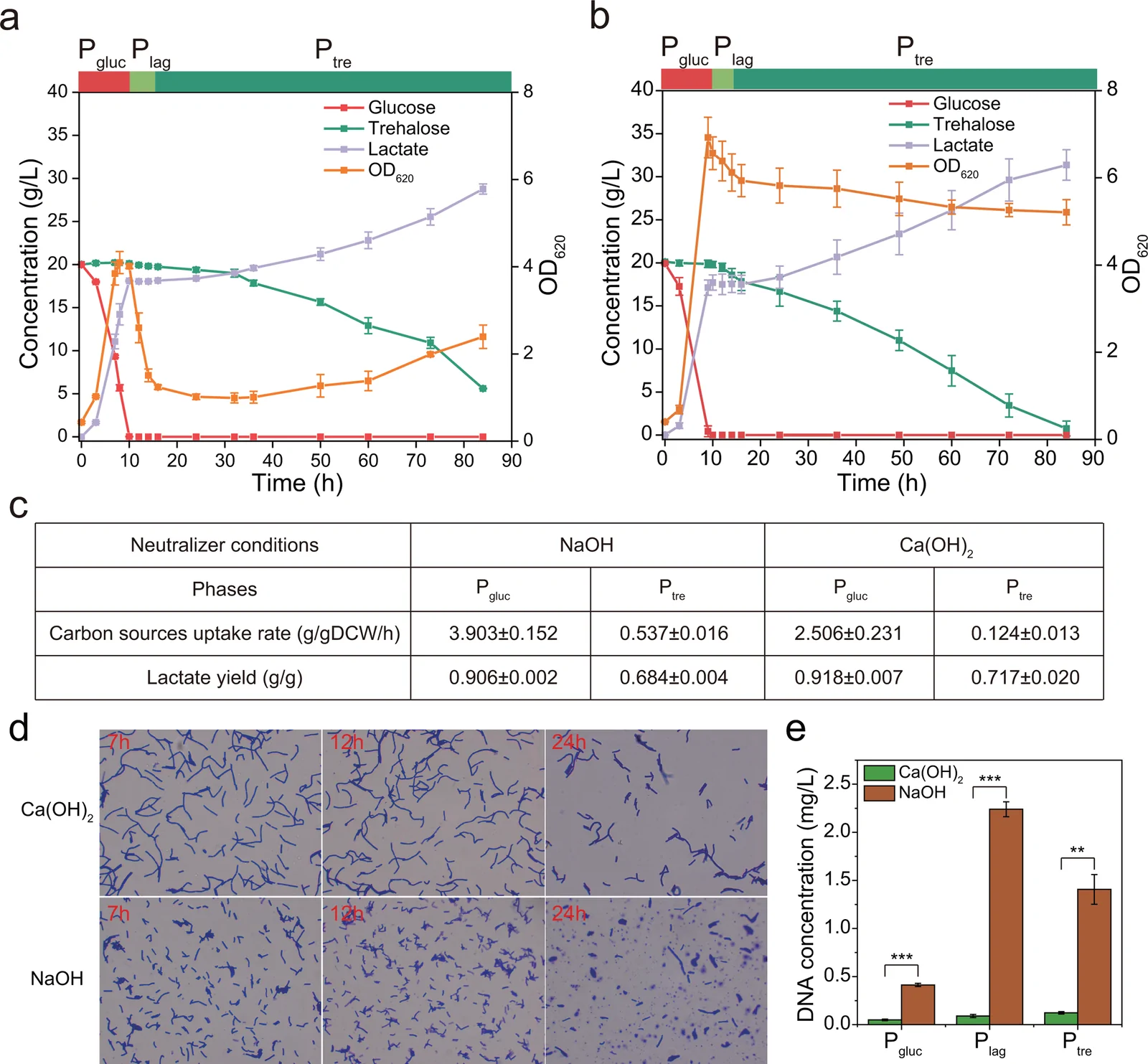
Fermentation results of mixed carbon sources under different neutralizer conditions in a 5 L bioreactor by *B. coagulans*. Fermentation processes on the combination of glucose and trehalose under sodium hydroxide (NaOH) (**a**) and calcium hydroxide (Ca(OH)_2_) (**b**). The results are averaged from three biological replicates. (**c**) Effect of neutralizer on fermentative parameters under different carbon source phases. (**d**) Microscopic morphology of cells at different phases. The figure shows the cell morphology under crystal violet staining, as observed with a 100× oil immersion lens. (**e**) Measurement of DNA concentration in the supernatant of fermentation broth at different phases. Statistical analysis between the two neutralizers was performed using a two-sided *t*-test. Statistically significant differences are described as follows: ***FDR < 0.001, **FDR < 0.01. The results are averaged from three biological replicates. P_gluc_, P_lag_, and P_tre_ denote the three phases of the fermentation process: glucose consumption, lactate production lag, and trehalose consumption, respectively.

*Bacillus coagulans* is a dominant strain in industrial lactate fermentation (20, 21), and starch-based materials containing various carbon sources are commonly used as substrates (22). Our previous work explored the batch fermentation of *Bacillus coagulans* DSM 1 = ATCC 7050 on mixed carbon sources (glucose and trehalose), and the fermentation process could be divided into the glucose phase (P_gluc_), lactate production lag phase (P_lag_), and trehalose phase (P_tre_) (Fig. 1) (19, 23). In this work, the fermentation processes were maintained at pH 6.0 using either sodium hydroxide (NaOH) or calcium hydroxide [Ca(OH)_2_], and the two neutralizers exhibited distinct phenotypic outcomes. In P_gluc_, the maximum OD_620_ measured under NaOH condition was 4.1 (Fig. 1a), which was substantially lower than the 6.6 measured under Ca(OH)_2_ conditions (Fig. 1b). However, the glucose uptake rate at NaOH was higher than that at Ca(OH)_2_ (Fig. 1c). Meanwhile, the lactate yields under the two conditions were quite similar, with the one from NaOH being slightly lower. Microscopic observation revealed that cells under sodium hydroxide conditions exhibited a shorter and more dispersed morphology compared with the typical chain-like structures observed under calcium hydroxide conditions (Fig. 1d), consistent with the known behavior of *B. coagulans* altering its cellular morphology under stress conditions (24). P_lag_ exhibited the most pronounced discrepancy in terms of biomass concentration. Although the decrease in OD during the carbon source transition has been observed in *Bacillus coagulans* (25–27), we found that the decrease in OD_620_ was more pronounced under the NaOH condition than under the Ca(OH)_2_ condition, with the OD_620_ dropping to 1.2 (Fig. 1a). In this phase, both microscopic observation (Fig. 1d) and visual inspection of the bioreactor (Fig. S1) revealed the presence of cellular debris. Furthermore, by measuring the DNA content in the fermentation supernatant, we found that the DNA concentration under NaOH conditions was significantly higher than that under Ca(OH)_2_ conditions, with the concentration at 12 h being nearly 25 times greater (Fig. 1e). These lines of evidence collectively indicated that cell lysis occurs following glucose depletion. In P_tre_, the trehalose uptake rate under NaOH conditions was higher, but the very small biomass concentration caused more residual trehalose, and the lactate yield was only 0.684 g/g, lower than the 0.717 g/g obtained under Ca(OH)_2_ conditions (Fig. 1c). At 84 h of fermentation, trehalose was nearly depleted under Ca(OH)_2_ conditions, with a total lactate production reaching 31.4 g/L. In contrast, under NaOH conditions, 5.6 g/L of trehalose remained, and the total lactate production was only 28.8 g/L. Although the sugar consumption rate was higher under NaOH conditions, the lower biomass resulted in a prolonged overall fermentation time. Taken together, these results highlight the impact of neutralizers on cell growth, carbon source utilization, and product production.

### Sodium ion stress is a key factor for the significant biomass decline during the shift of carbon sources

In order to explore the reason for biomass decline, we used the mixture of Ca(OH)_2_ and NaOH as a neutralizer to control the pH (Fig. S2a). Notably, the biomass was still reduced for a period of time after glucose depletion, indicating that the addition of NaOH led to cell lysis. The rate of lysis was slower compared to the condition with sodium hydroxide alone, which may be attributed to the protective effect of calcium hydroxide on the cells. We further examined whether cellular lysis under NaOH conditions resulted from depletion of other nutrients in the medium. However, the biomass still decreased significantly when complex medium (28) was used (Fig. S2b). Although biomass was slightly higher than that observed in the synthetic medium and cell lysis was delayed, possibly due to the presence of complex components that provide a weak protective effect against stress, the biomass reduction remained very pronounced compared to that under Ca(OH)_2_ conditions. Furthermore, extracellular amino acid measurements confirmed these components remained abundant after glucose depletion (Fig. S3a). These findings collectively demonstrate that nutrient exhaustion alone is not the primary driver of lysis, although it may act as a contributing stressor during the carbon source transition.

**Fig 2:**
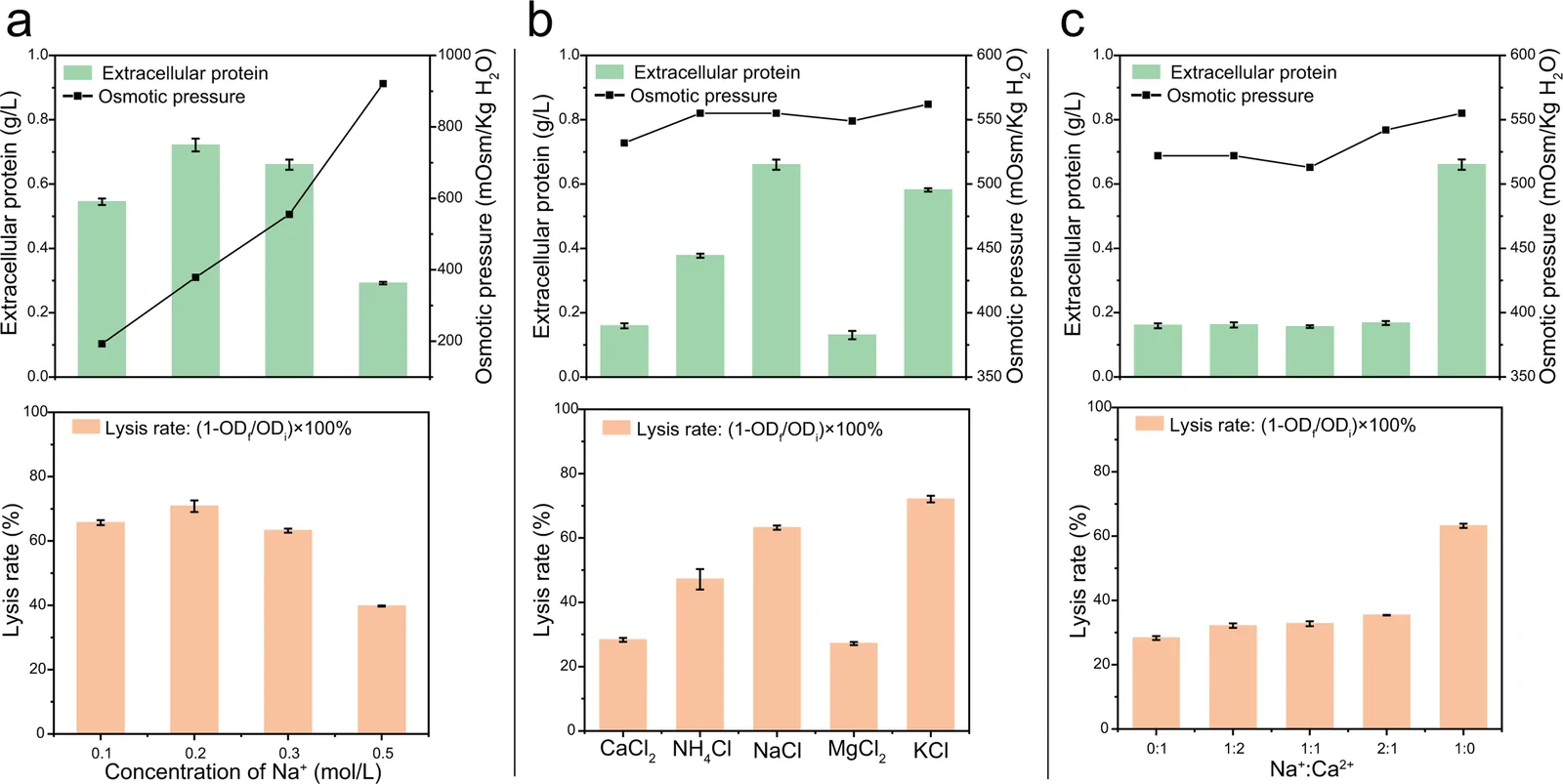
Investigation of cell lysis factors. Effects of different ion conditions on cell lysis: (**a**) different Na^+^ concentration; (**b**) different metal ions; the total concentration of anions and cations is 0.6 mol/L; (**c**) different Na^+^:Ca^2+^ mole ratio; the total concentration of anions and cations is 0.6 mol/L. The lysis rate is defined as the ratio of the reduced OD (OD_i_−OD_f_) after 12 h in the metal ion solution to the initial OD (OD_i_), multiplied by 100%. The results are averaged from three biological replicates.

We next evaluated whether osmotic pressure contributed to biomass decline. Under calcium hydroxide and sodium hydroxide conditions, the osmotic pressures of the fermentation broths after glucose depletion were 500 and 550 mOsm/kg, respectively, with calcium ion and sodium ion concentrations of 0.11 and 0.19 mol/L, respectively (Fig. S3b). Cells harvested at 10 h under Ca(OH)_2_ conditions were resuspended in sodium chloride solutions of varying concentrations and incubated for 12 h to simulate carbon source depletion under different osmotic pressures. Notably, the extent of cell lysis and protein release was lower at 0.5 mol/L NaCl, whereas these parameters were increased at concentrations approximating the osmotic pressure of the fermentation broth (0.1–0.3 mol/L NaCl) (Fig. 2a). This observation suggested that isotonic conditions prevent cell swelling and rupture, whereas hypotonic conditions exacerbate cell disruption by promoting water influx.

To further determine whether the observed lysis was ion-specific rather than a general osmotic effect, cells were resuspended in solutions containing different metal ions while maintaining comparable osmotic pressure to that observed during fermentation. The cell lysis rate was significantly higher in monovalent ion solutions (such as sodium and potassium) than in divalent ion solutions (such as calcium and magnesium) (Fig. 2b), which was consistent with reports that sodium ions can induce cell lysis (29), whereas calcium and magnesium ions play a role in maintaining cell wall integrity and preventing lysis (30, 31). Subsequently, cells were incubated in solutions with varying sodium-to-calcium ion concentration ratios. The presence of calcium ions significantly reduced cell lysis compared to Na^+^-only solutions (Fig. 2c), demonstrating a protective effect of calcium ions. Even in the presence of calcium ions, the addition of sodium ions still increased cell lysis, as observed with the mixed neutralizer (Fig. S2a). This suggested that sodium ions counteract the protective effect of calcium ions, possibly through competitive binding with cell wall components (30, 32).

Collectively, these results, obtained under carbon-free conditions that recapitulate the physiological state of cells during carbon source transition, indicate that extensive cell lysis is ion-specific. Compared with calcium ions, sodium ions render cells more sensitive to hypotonic conditions, which we attribute to sodium-induced cell wall damage in the absence of an energy source, whereas calcium ions exert a protective effect. Accordingly, when Ca(OH)_2_ was used as a neutralizer, sodium stress was avoided, and additional calcium-mediated protection was provided, enabling cells to maintain higher stability during carbon source transition.

### Metabolomic analysis under different neutralizer conditions

**Fig 3:**
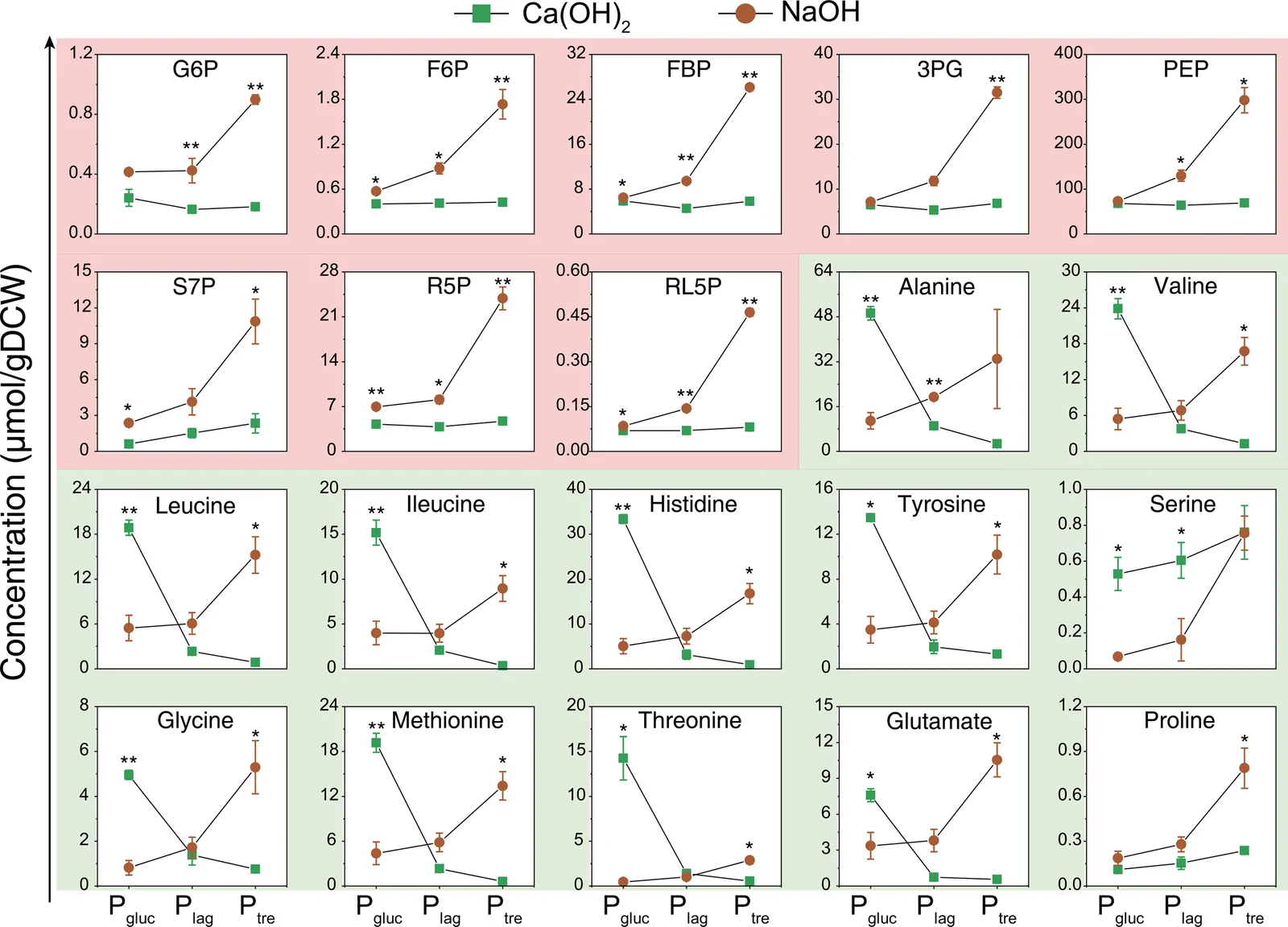
Concentrations of intracellular metabolites under different neutralizer conditions. The phosphate sugars and amino acids from the three phases are taken from 7, 12, and 48 h of the fermentation process. Data in the bar diagram are mean ± s.d. of three biological replicates. Statistical analysis between the two neutralizers was performed using a two-sided *t*-test. Statistically significant differences are described as follows: **FDR < 0.01, *FDR < 0.05. The descriptions of metabolite abbreviations are provided in the supplemental material.

To investigate metabolic differences between the two neutralizer conditions, we measured intracellular metabolite concentrations at 7, 12, and 48 h, representing the P_gluc_, P_lag_, and P_tre_ phases for comparison. Comparative analysis of intracellular phosphorylated sugar concentrations revealed that during P_gluc_, most metabolites in the glycolysis pathway, such as glucose-6-phosphate (G6P), fructose-6-phosphate (F6P), fructose-1,6-bisphosphate (FBP), 3-phosphoglycerate (3PG), and phosphoenolpyruvate (PEP), exhibited minor or insignificant differences between the two neutralizer conditions (Fig. 3). This suggested that glucose metabolism was largely unaffected by neutralizer choice when glucose was abundant and growth demand was high. Notably, metabolites of the phosphoketolase pathway, such as sedoheptulose-7-phosphate (S7P), ribose-5-phosphate (R5P), and ribulose-5-phosphate (RL5P), all showed significantly elevated levels during P_gluc_ under NaOH conditions compared to Ca(OH)_2_. This indicated enhanced activation of the phosphoketolase pathway to increase ATP yield (33), which concomitantly reduces lactic acid production, consistent with fermentation performance data (Fig. 1c). After glucose depletion, intracellular phosphorylated sugar levels increased markedly under NaOH conditions, with concentrations in P_tre_ exceeding those in P_gluc_. This may reflect a cellular strategy for energy storage under stress (34). Concurrently, limitations in lactate production at the downstream end of the glycolytic pathway could also result in the accumulation of these metabolites (35), thereby driving metabolic redistribution that allocates carbon into other pathways, such as amino acid metabolism, in response to environmental stress (36, 37).

During P_gluc_, most intracellular amino acid concentrations were significantly higher under Ca(OH)_2_ conditions than under NaOH (Fig. 3), indicating more active amino acid metabolism and biomass synthesis. This trend was consistent with extracellular amino acid profiles (Fig. S3a), where Ca(OH)_2_-supported cultures consumed amino acids more rapidly to meet higher growth demands. After glucose depletion, intracellular amino acid levels declined sharply under Ca(OH)_2_ conditions but remained stable or slightly increased under NaOH, resulting in no significant difference between the two conditions. However, extracellular amino acid measurements during P_lag_ indicated active uptake under Ca(OH)_2_, reflecting proteome reallocation, whereas NaOH cultures showed little net uptake, likely due to amino acid release from lysed cells. As trehalose utilization progressed, intracellular amino acid concentrations under NaOH conditions increased substantially and exceeded both Ca(OH)_2_ levels and NaOH P_gluc_ levels. This accumulation is likely attributable to intracellular protein degradation. Consistent with previous reports that elevated intracellular amino acid pools can mitigate sodium lactate stress (38), amino acids may partially contribute to stress resistance and support limited growth recovery during P_tre_.

### Identification of differentially expressed genes between different neutralizer conditions

To elucidate the transcriptional basis underlying the metabolic differences induced by different neutralizers, transcriptome analysis was performed using samples collected at 7, 12, and 48 h. Principal component analysis (Fig. S4a) and Pearson correlation analysis (Fig. S4b) revealed great consistency among biological replicates and clear separation among fermentation phases, indicating robust data quality. Within each neutralizer condition, P_gluc_ and P_tre_ samples clustered closely, whereas P_lag_ samples were distinctly separated from the other two phases.

**Fig 4:**
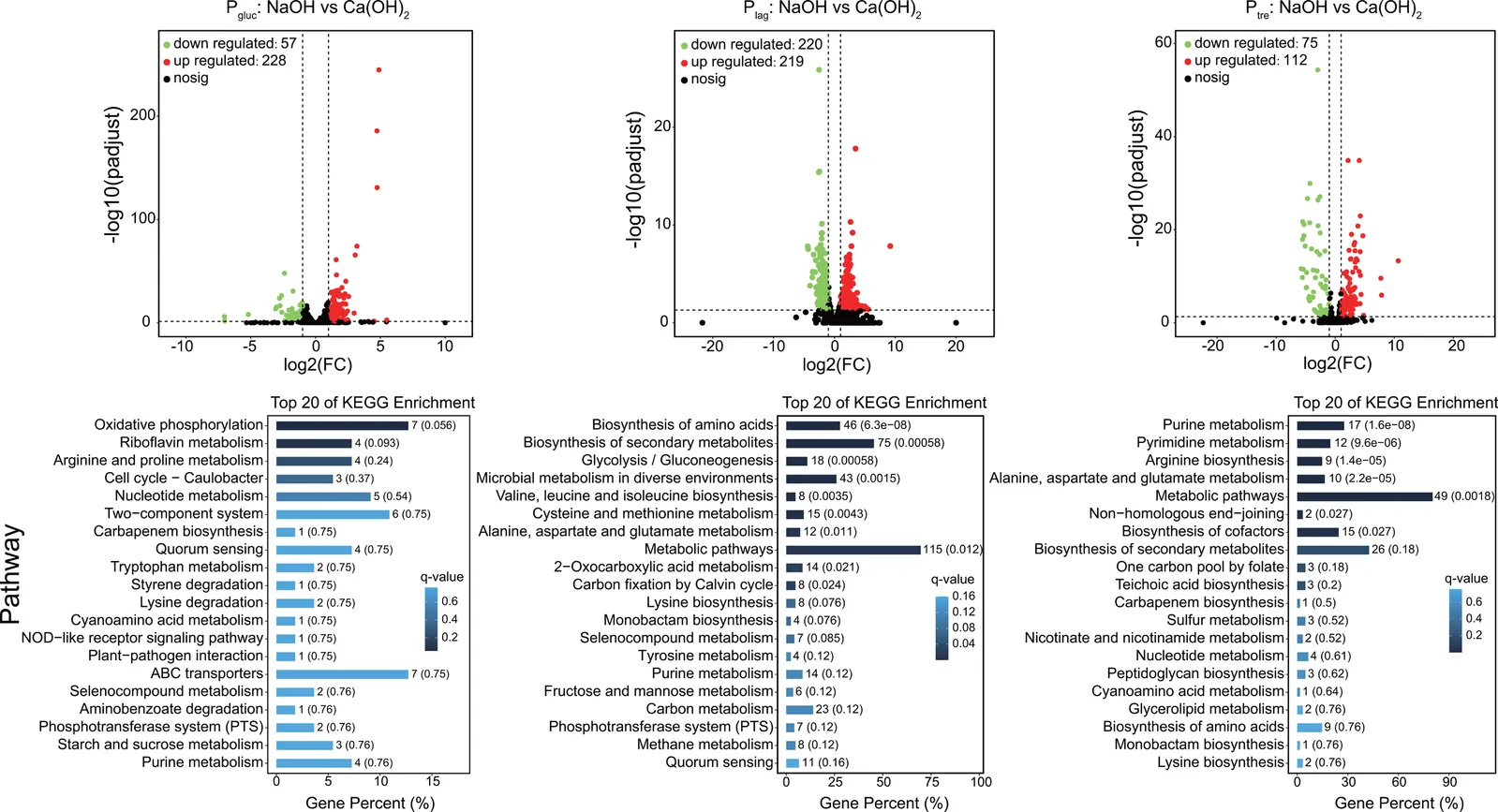
Differential analysis of transcriptome between different neutralizer conditions. The transcriptomic data from the three phases are taken from 7, 12, and 48 h of the fermentation process. The panel above shows the volcano plot of the differential genes of NaOH versus Ca(OH)_2_, and the panel below shows the pathways of the top 20 of the corresponding KEGG enrichment analysis. The numbers following each enriched pathway represent the percentage of differentially expressed genes enriched in that pathway and the corresponding q-value, respectively.

Notably, P_lag_ samples under NaOH and Ca(OH)_2_ conditions were clearly separated, suggesting substantial transcriptional divergence during the carbon source transition phase, likely associated with extensive cell lysis. Differentially expressed genes (DEGs) were identified using thresholds of |log_2_FC| > 1 and *P*_adjust_ < 0.05 (Fig. 4). At P_gluc_, NaOH treatment resulted in 57 downregulated and 228 upregulated genes compared with Ca(OH)_2_. At P_lag_, 220 genes were downregulated and 219 were upregulated, representing the most extensive transcriptional remodeling among the three phases. At P_tre_, 75 genes were downregulated and 112 were upregulated. To determine the pathways impacted by neutralizer selection, KEGG enrichment analysis was carried out (Fig. 4). At P_gluc_, no pathway was significantly enriched, which was in line with the limited phenotypic differences observed at this phase. At P_lag_, DEGs were significantly enriched in glycolysis/gluconeogenesis and multiple amino acid metabolism pathways, all of which were downregulated under NaOH conditions. This global suppression of metabolic pathways corresponds to the severe biomass loss observed during P_lag_ (Fig. 1a). Interestingly, the downregulation of amino acid biosynthesis pathways contrasted with elevated intracellular amino acid concentrations, suggesting regulation beyond transcription, such as enzyme activity control (39) or amino acid release from lysed cells. At P_tre_, DEGs were enriched in purine metabolism, pyrimidine metabolism, and several amino acid metabolism pathways. The majority of these pathways were downregulated, but a small subset was upregulated, which would support partial growth recovery.

### Insufficient energy supply under sodium stress leads to cellular lysis

**Fig 5:**
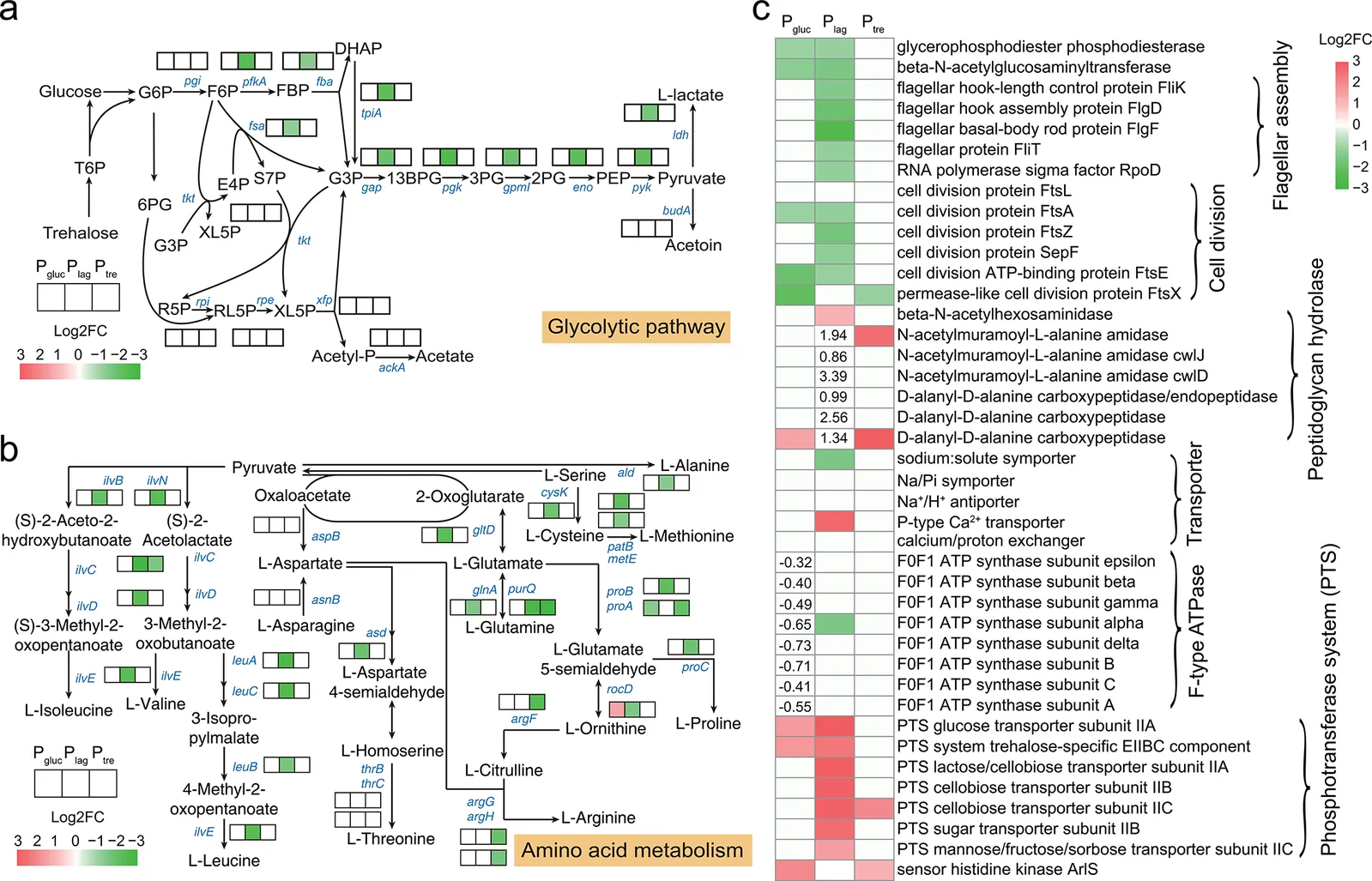
Comparison of the differences in transcriptome between different neutralizer conditions. Heatmap of the glycolytic pathway (**a**), amino acid metabolism (**b**), and some key differentially expressed genes (**c**) in three phases of fermentation. Heatmap colors indicate gene expression levels (log_2_FC) of sodium hydroxide compared to calcium hydroxide. The descriptions of metabolite abbreviations are provided in the supplemental material.

As shown in Fig. 5a, genes involved in glycolysis exhibited significant downregulation during P_lag_ under NaOH conditions, while remaining largely unchanged during P_gluc_ and P_tre_. This indicated that neutralizer-induced disruption of energy metabolism is phase-dependent and becomes critical during carbon source transition. A similar trend was observed for amino acid metabolism (Fig. 5b). During P_lag_, genes involved in the biosynthesis of valine, leucine, isoleucine, alanine, glutamate, cysteine, methionine, and proline were significantly downregulated. These amino acids are known to contribute to stress tolerance; therefore, their reduced biosynthetic capacity suggests that sodium stress exceeded the compensatory limits of amino acid-mediated defense mechanisms. Several stress-related and ion transport genes also exhibited marked expression changes (Fig. 5c). Under sodium stress, sodium:solute symporters were significantly downregulated, likely representing an adaptive strategy to limit further Na^+^ influx. In contrast, Na^+^/H^+^ antiporters (40) showed only marginal and non-significant upregulation, suggesting a secondary role in sodium extrusion. F-type ATP synthase genes were significantly downregulated during P_gluc_ under NaOH conditions. Although this enzyme supports ATP synthesis, it can also mediate sodium transport through ATP hydrolysis. Downregulation of ATP synthase likely restricts Na^+^ uptake but simultaneously suppresses ATP generation (41), increasing dependence on carbon source-derived energy. Consistent with this interpretation, genes encoding phosphotransferase system (PTS) transporters were strongly upregulated, indicating enhanced carbon uptake to meet elevated ATP demand (42).

Meanwhile, purine metabolism genes were significantly downregulated during P_lag_ (Fig. S5), further reflecting cellular energy deficiency. Genes encoding dehydrogenases involved in fatty acid degradation were upregulated, suggesting increased acetyl-CoA supply to support energy metabolism. Conversely, genes involved in membrane and cell wall biosynthesis, cell division, and flagellar assembly were markedly downregulated. Because sodium ions are known to drive flagellar motility (43), repression of flagellar genes may further reduce sodium influx. However, when sodium extrusion capacity is exceeded and ATP supply becomes insufficient, cells initiate autolysis (29). We also observed upregulation of the gene encoding histidine kinase ArlS in the two-component system, which senses environmental stress. This protein transmits environmental signals to downstream regulatory genes via a phosphorylation mechanism (44, 45). It has been reported that these genes are involved in spore formation (46, 47) and peptidoglycan hydrolase production (48, 49) under stress conditions. In our study, multiple peptidoglycan hydrolases genes were strongly upregulated in P_lag_ under NaOH conditions. This finding is consistent with studies showing that under high-sodium chloride stress, peptidoglycan synthesis is inhibited while peptidoglycan hydrolases are upregulated, leading to cell lysis (50). This response suggests a population-level survival strategy, whereby lysis of a large fraction of cells releases nutrients to sustain a smaller surviving subpopulation (51).

Under Ca(OH)_2_ conditions, genes encoding P-type calcium transporters were downregulated, indicating active regulation of intracellular Ca^2+^ homeostasis. Unlike NaOH treatment, Ca(OH)_2_ did not inhibit cell growth, likely due to the protective effects of calcium ions. Calcium ions interact with phospholipid components of the cell wall, enhancing cross-linking and improving structural stability (52). Additionally, calcium contributes to anchoring cell surface proteins (30) and stimulates heat shock protein activity, thereby enhancing thermal resistance (53, 54). For thermophilic *B. coagulans* cultivated at 50°C, maintaining cellular integrity under carbon-limited conditions is critical for survival. Previous studies have shown that calcium supplementation improves the viability of lactic acid bacteria during stress conditions, such as lyophilization (53, 55, 56). Together, these effects explain the superior stability of cells under Ca(OH)_2_-neutralized fermentation.

To compare the energy status under the two conditions, we determined the ratio of intracellular ATP to ADP concentrations (Fig. S6). The results showed that under the NaOH condition, the ATP/ADP ratio in the P_gluc_ was higher than that under the Ca(OH)_2_ condition. This suggests that under sodium ion stress, cells require a higher energy status to simultaneously support growth and cope with the stress, which may explain the lower biomass observed under this condition (Fig. 1). Interestingly, in the P_lag_ phase, the ATP/ADP ratio under the NaOH condition remained significantly higher than that under the Ca(OH)_2_ condition. This is because the surviving cells still require a high-energy state to resist stress. However, due to carbon source depletion during this phase, not all cells can sustain such a high-energy status, leading to extensive cell lysis with only a small subpopulation of cells surviving.

### Transcriptional trend analysis reveals neutralizer-specific adaptive strategies during carbon source transition

**Fig 6:**
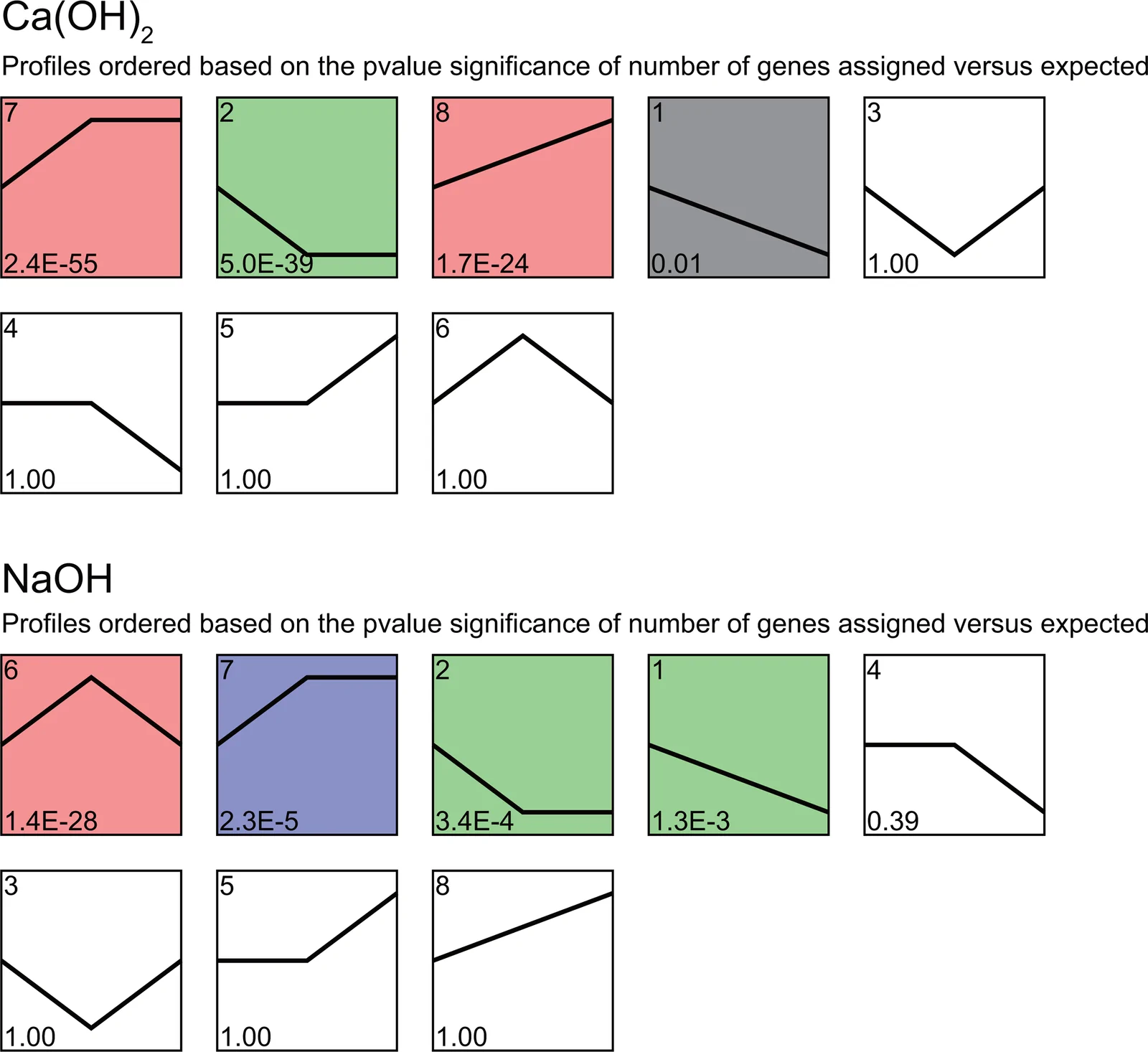
Transcriptional trend analysis across fermentation phases under Ca(OH)_2_ and NaOH neutralization. Eight expression clusters were generated per condition based on gene expression change across three phases (P_gluc_, P_lag_, P_tre_). Colored clusters indicate significant enrichment (*P* < 0.05). Genes with a log_2_FC greater than 1 are considered changed genes.

To investigate dynamic transcriptional responses across the three fermentation phases (P_gluc_, P_lag_, P_tre_), we performed trend analysis under each neutralizer condition. Under both Ca(OH)_2_ and NaOH, four significant gene clusters (*P* < 0.05) were identified (Fig. 6). Three of these clusters (clusters 7, 2, and 1) exhibited similar expression trends between the two neutralizers, indicating shared transcriptional programs during carbon source transition. The key difference between conditions lay in one unique cluster: Cluster 8 under Ca(OH)_2_ and cluster 6 under NaOH (Fig. 6).

KEGG enrichment analysis of these clusters (Fig. S7) revealed distinct functional strategies. In cluster 7 (shared), enriched pathways were largely similar between neutralizers, suggesting limited sensitivity to neutralizer type. However, under NaOH conditions, the non-homologous end-joining pathway was uniquely enriched, primarily involving DNA repair genes, consistent with stress-induced cellular damage. Under Ca(OH)_2_ conditions, flagellar assembly pathways were enriched in clusters 2 and 1, while ribosome-related pathways were enriched in cluster 2. These trends indicated growth attenuation during carbon source transition, consistent with the observed growth plateau (Fig. 1a). Under NaOH conditions, amino acid biosynthesis pathways were enriched in cluster 2 despite rising intracellular amino acid levels (Fig. 3). This discrepancy suggested that amino acid accumulation originated primarily from protein degradation rather than *de novo* synthesis and may also reflect feedback inhibition or energy conservation (57). In cluster 1, ribosome pathway enrichment indicated global suppression of protein synthesis, allowing resource prioritization toward stress response (58, 59).

The most informative differences emerged from the unique clusters. In cluster 8 under Ca(OH)_2_ conditions, arginine biosynthesis and oxidative phosphorylation pathways were significantly enriched. Arginine metabolism generates ammonia, contributing to pH stabilization (60), while enhanced oxidative phosphorylation supports energy demands during trehalose utilization (23). In cluster 6 under NaOH conditions, enriched pathways were predominantly associated with carbon metabolism and energy production. Notably, these pathways were transiently activated following glucose depletion and subsequently downregulated once trehalose metabolism was established, reflecting a short-lived compensatory response rather than a sustainable adaptation. Furthermore, we found numerous sporulation protein genes and peptidoglycan hydrolase genes within this trend, which was consistent with the transcriptional comparison results relative to Ca(OH)_2_.

Taken together, the transcriptional trend analysis revealed that while both neutralizers trigger growth attenuation during carbon source transition, NaOH uniquely induces a transient surge in carbon source transport and metabolism, energy metabolism, and cell lysis. These characteristics indicated that sodium hydroxide imposed a more severe and energetically demanding environment compared to calcium hydroxide throughout the mixed-carbon-source fermentation process.

## DISCUSSION

This study demonstrates that the choice of pH neutralizer critically influences both fermentation performance and cell survival of *Bacillus coagulans* during batch fermentation with mixed carbon sources. Specifically, the use of sodium hydroxide induces severe cell lysis upon glucose depletion, whereas calcium hydroxide maintains cellular integrity throughout the fermentation process. Physiological characterization and ion-controlled experiments revealed that sodium ion stress was the main cause of this cell lysis. Although sodium hydroxide effectively maintains extracellular pH, the continuous accumulation of sodium ions imposes a substantial energetic burden on cells, forcing them to activate energy-intensive stress resistance mechanisms. Integrated metabolomic and transcriptomic analyses further revealed that sodium ion stress drives cells into a high-energy-consumption survival state. Under NaOH conditions, cells downregulated growth-associated processes and sodium uptake systems while simultaneously upregulating carbon source transporters to compensate for increased ATP demand. This regulatory strategy enables cells to temporarily withstand sodium stress when glucose is abundant.

**Fig 7:**
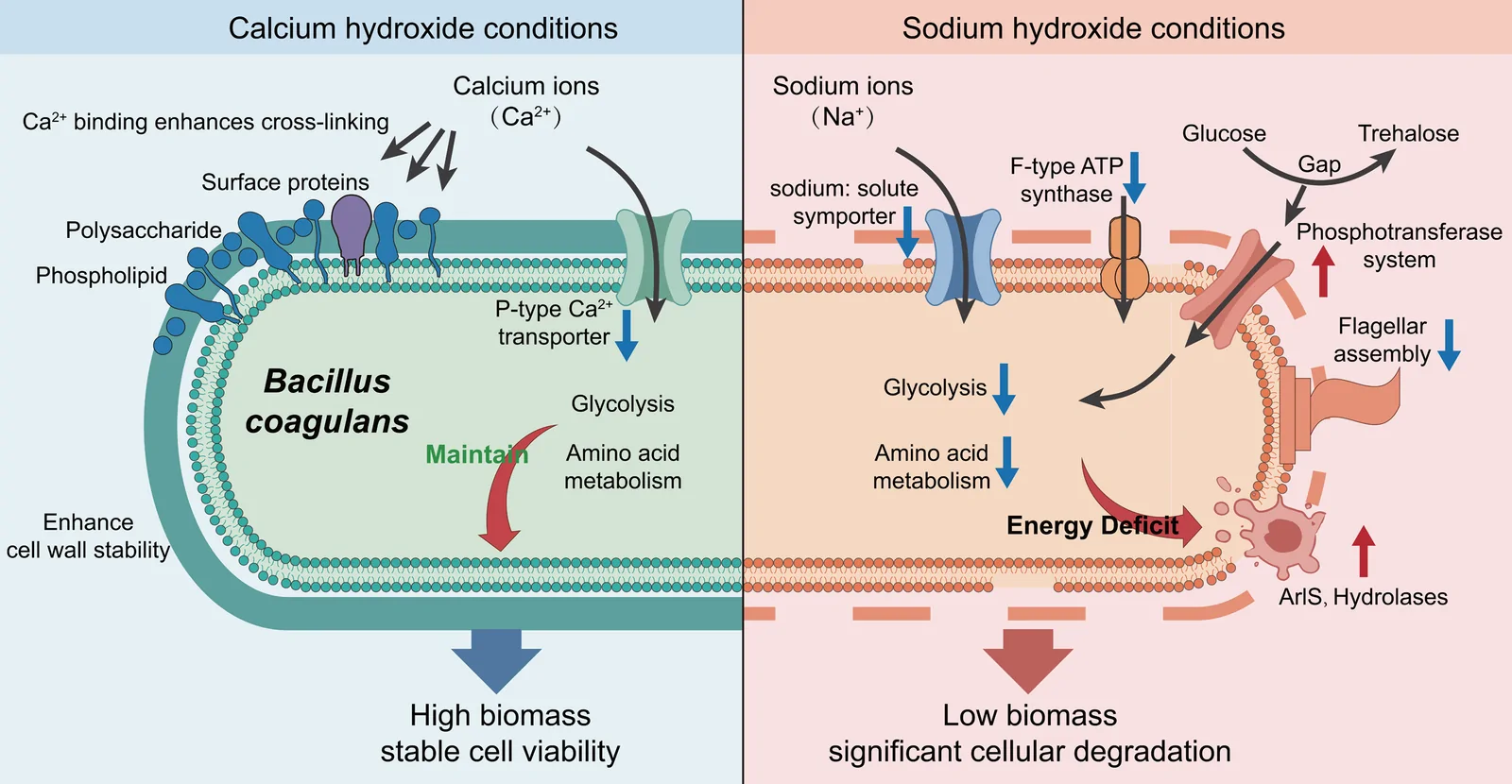
Cellular metabolic response mechanisms of calcium hydroxide and sodium hydroxide as neutralizers in mixed carbon source lactic acid fermentation of *Bacillus coagulans*. Upward red arrows indicate upregulated genes or pathways, while downward blue arrows denote downregulated genes or pathways.

However, during the critical carbon source transition phase, this balance collapses. Glucose depletion necessitates extensive proteome and metabolic reallocation toward trehalose utilization. During this transition, carbon uptake is transiently insufficient, leading to stagnation of glycolysis and amino acid metabolism. As a result, ATP generation fails to meet the energetic requirements imposed by sodium ion extrusion and stress resistance. The resulting energy deficit ultimately triggers a programmed-like autolytic response. Upregulation of sensor histidine kinase ArlS and multiple peptidoglycan hydrolases promotes large-scale cell lysis, releasing intracellular nutrients to support the survival of a smaller subpopulation. This phenomenon reflects a population-level survival strategy under extreme energetic stress rather than a random collapse of cellular regulation. In contrast, calcium hydroxide avoids sodium ion stress entirely and provides additional protective effects. Calcium ions enhance cell wall stability through interactions with phospholipids and polysaccharides and contribute to the anchoring of surface proteins. Moreover, calcium has been shown to stimulate heat shock protein activity and improve thermotolerance, which is particularly relevant for thermophilic *B. coagulans* cultivated at elevated temperatures (Fig. 7).

Although minor biomass decline was still observed during carbon source transition under Ca(OH)_2_ conditions, this phenomenon has been widely reported in *B. coagulans* (25, 26) and other microorganisms (61, 62). It likely reflects delayed carbon uptake during metabolic switching rather than stress-induced cell lysis. Notably, in laboratory-evolved (Fig. S8) and engineered strains (22, 63, 64) capable of overlapping utilization of multiple carbon sources, biomass decline during transition was eliminated, further supporting the central role of carbon uptake continuity. These observations highlight that carbon source transition represents a vulnerable window during mixed-carbon-source fermentation. When additional stresses, such as sodium ion accumulation, coincide with this transition, cellular energy homeostasis can be rapidly disrupted, leading to catastrophic loss of biomass.

From an industrial perspective, maintaining effective cell density during the late fermentation stage is critical for efficient utilization of residual sugars, such as trehalose (65). Excessive cell lysis not only reduces lactic acid productivity but also prolongs fermentation time and decreases process robustness. Therefore, neutralizer selection emerges as a crucial yet often underestimated parameter in industrial lactic acid fermentation processes.

### Conclusion

In summary, this study elucidates the cellular response mechanisms of *Bacillus coagulans* to sodium hydroxide and calcium hydroxide as pH neutralizers during mixed-carbon-source lactic acid fermentation. When sodium hydroxide is used, substantial cell lysis occurs during the carbon source transition phase. This cell lysis is primarily driven by the combined effects of sodium ion stress and insufficient energy supply. Sodium ion accumulation imposes a high energetic burden on cells, and during the transition from glucose to trehalose utilization, transient carbon uptake limitation leads to impaired glycolysis and amino acid metabolism. The resulting energy deficit ultimately triggers the upregulation of autolytic enzymes, causing large-scale population collapse.

In contrast, calcium hydroxide does not introduce sodium ion stress and provides additional protective effects that stabilize cell structure and maintain cellular integrity during carbon source transition. As a result, higher cell density is preserved, enabling more efficient utilization of residual carbon sources and improved fermentation performance. Overall, these findings demonstrate that neutralizer selection plays a critical role in determining both metabolic stability and process efficiency in mixed-carbon-source lactic acid fermentation. This work provides a mechanistic basis for rational neutralizer selection and offers valuable guidance for fermentation process optimization and the development of stress-resistant industrial strains.

## MATERIALS AND METHODS

### Microorganism, media, and culture conditions

*B. coagulans* DSM 1 = ATCC 7050 was purchased from the American Type Culture Collection.

Seed cultures were prepared in MRS medium, and batch fermentations were conducted in chemically defined MCDM3++ medium (28) with a mixed carbon source of 20 g/L glucose and 20 g/L trehalose.

Seed cultivation was carried out in 250 mL shake flasks containing 100 mL working volume, incubated at 50°C and 100 rpm, with pH buffered by calcium carbonate. After approximately 12 h, cells were harvested, washed twice with 100 mM phosphate buffer, resuspended in ultrapure water, and inoculated at 10% (vol/vol) into a 5 L bioreactor.

Batch fermentation was carried out in the 5 L bioreactor with a 4 L working volume under the following conditions: 50°C, 100 rpm, and an aeration rate of 7.2 L/h. The pH was automatically maintained at 6.0 by adding either 25% (wt/vol) calcium hydroxide or sodium hydroxide. All experiments were performed in triplicate.

### Determination of biomass, lactate, carbon sources, and DNA

Biomass concentration was determined by measuring the optical density (OD) at 620 nm using a spectrophotometer.

Lactate concentration in the fermentation broth was analyzed by high-performance liquid chromatography (HPLC; Shimadzu LC20AT) equipped with a Hi-Plex H column (300 mm × 7.7 mm). Detection was performed at 210 nm with the column temperature maintained at 50°C, using 0.01 mol/L sulfuric acid as the mobile phase at a flow rate of 0.4 mL/min.

Carbon sources in the fermentation broth were quantified using an ion chromatography system (Dionex ICS-3000) fitted with a Dionex Carbopac PA20 column (65). Prior to analysis, samples were diluted to ensure sugar concentrations fell within the detection range. Calcium ions in the medium were precipitated with saturated sodium oxalate solution, and proteins were removed by ethanol treatment. The processed solution was then evaporated to obtain solid sugars, which were subsequently redissolved in water for analysis. The elution procedure was performed using ultrapure water and 200 mmol/L NaOH at a ratio of 9:1 for 18 min, followed by elution at a ratio of 2:8 for 17 min, and finally re-equilibration at 9:1 for 35 min, with a constant flow rate of 0.4 mL/min maintained throughout the process.

Extracellular DNA concentration was determined using the Quantus Fluorometer method. Fermentation broth samples and DNA standards at various dilutions were each mixed with an equal volume of PicoGreen fluorescent dye and incubated for 5 min in the dark. Fluorescence was then measured using a Quantus Fluorometer, and the DNA concentration in the fermentation broth was calculated based on a standard curve.

### Determination of intracellular metabolites

The metabolome samples were collected at 7, 12, and 48 h, corresponding to P_gluc_, P_lag_, and P_tre_, respectively. Intracellular metabolites were quantified using isotope dilution mass spectrometry (IDMS) in selected ion monitoring mode (66). Prior to extraction, a known concentration of U-^13^C-labeled cell extracts was added as internal standards and mixed with the collected cells. This approach maintains a constant ratio between intracellular ^12^C-metabolite and ^13^C-metabolite concentrations throughout extraction, derivatization, and detection, thereby minimizing systematic errors and improving data reliability.

Absolute quantification of intracellular phosphate sugars and amino acids was carried out via gas chromatography-mass spectrometry (Agilent, GC-7890A, MS-5975C) (67, 68). Cell metabolism was quenched before metabolite extraction by rapidly mixing 2 mL of fermentation broth with 30 mL of 60% methanol pre-cooled to −27°C in a 50 mL centrifuge tube. The cells were quenched for 5 min, and then 100 μL of U-^13^C-labeled cell extracts were added as internal standards. Seventy-five percent ethanol solution preheated to 80°C was added to the collected cells and boiled for 5 min. The resulting liquid was concentrated to 600 μL by rapid evaporation, and 100 μL of this concentrate was taken as the sample for freeze-drying prior to derivatization.

Amino acid derivatization method: the freeze-dried sample was mixed with 100 μL pyridine and dissolved at 65°C for 1 h. After cooling to room temperature, 100 μL of derivatization reagent (N‐tert‐butyldimethylsilyl‐N-methyl-trifluoroacetamide with 1% tert‐butyldimethylchlorosilane) was added. Derivatization was then performed at 65°C for 1 h. Subsequently, the sample was centrifuged at 12,000 rpm for 5 min, and the supernatant was analyzed by GC-MS.

Phosphate sugars derivatization method: the freeze-dried sample was treated with 50 μL of a 20 mg/mL methoxyamine solution in pyridine and incubated at 65°C for 1 h. After cooling to room temperature, 80 μL of N-methyl-N-(trimethylsilyl) trifluoroacetamide/trimethylchlorosilane (MSTFA/TMCS, 1,000:50, vol/vol) was added, followed by derivatization at 65°C for 1 h. Subsequently, the sample was centrifuged at 12,000 rpm for 5 min, and the resulting supernatant was subjected to GC-MS analysis.

The GC-MS detection conditions (68) were as follows: the interface temperature was maintained at 280°C, the transfer line at 250°C, and electron impact ionization was performed at 230°C. The quadrupole temperature was set to 150°C, and the injection volume was 1 μL. Separation was carried out on an HP-5MS column (30 mm × 0.25 mm × 0.25 µm). For amino acids, the temperature program consisted of an initial column temperature of 100°C for 1 min, then an increase at a rate of 10°C/min to 320°C, which was held for 10 min. The temperature program for phosphate sugars began with a column temperature of 70°C for 1 min. After that, it increased at a rate of 10°C per minute until it reached 300°C, which it then held for 10 min.

The quantification of intracellular energy metabolites was also performed using the IDMS method with detection by LC-MS/MS. The specific details of the LC-MS/MS analytical procedure employed have been described in previous publications (69).

### Transcriptome analysis

The transcriptome samples were collected at 7, 12, and 48 h, corresponding to P_gluc_, P_lag_, and P_tre_, respectively. Cells were collected from fermentation broths under different neutralizer conditions and washed three times with phosphate-buffered saline to prepare for RNA extraction. Total RNA was isolated using TRIzol Reagent according to the manufacturer’s protocol, and genomic DNA contamination was removed by treatment with DNase I (TaKara). RNA quality was assessed on an Agilent 2100 Bioanalyzer, and concentration was measured using an ND-2000 spectrophotometer (NanoDrop Technologies).

For RNA library construction, the TruSeqTM RNA sample preparation Kit from Illumina (San Diego, CA) was employed. The rRNA was depleted using the Ribo-Zero Magnetic Kit. Double-stranded cDNA was then synthesized by reverse transcription with random primers using the SuperScript Double-Stranded cDNA Synthesis Kit (Invitrogen, CA). During second-strand synthesis, dUTP was substituted for dTTP. The resulting dUTP-containing second strand was subsequently digested to retain only the first cDNA strand. After amplification with Phusion DNA polymerase and quantification using TBS380, the library was sequenced on the Illumina NovaSeq platform with paired-end reads.

Bioinformatic analysis was performed on the resulting sequencing data. The reference genome and annotation files for *Bacillus coagulans* DSM 1 = ATCC 7050 were obtained from the National Center for Biotechnology Information. All RNA extraction and downstream analyses were conducted on the Majorbio Cloud Platform.

## Data Availability

The transcriptomics data have been deposited in the NCBI database under the BioProject accession number PRJNA1406494.

## SUPPLEMENTAL MATERIAL

Supplemental material aem.01314-26-s0001.docx Fig. S1 to S8.

ASM does not own the copyrights to Supplemental Material that may be linked to, or accessed through, an article. The authors have granted ASM a non-exclusive, world-wide license to publish the Supplemental Material files. Please contact the corresponding author directly for reuse.

## References

[1] Yu W, Zhang C, Li Y, Chen QA, Fei Q, Gao J, Zhou YJ. 2025. Methanol biotransformation for the production of biodegradable plastic monomer L-lactate in yeast. Nat Commun 16:10756. doi:10.1038/s41467-025-65793-x PMC12663439 41315207

[2] Zhang Z, Xie Y, He X, Li X, Hu J, Ruan Z, Zhao S, Peng N, Liang Y. 2016. Comparison of high-titer lactic acid fermentation from NaOH- and NH_3_-H_2_O_2_-pretreated corncob by *Bacillus coagulans* using simultaneous saccharification and fermentation. Sci Rep 6:37245. doi:10.1038/srep37245 PMC5112544 27853308

[3] de Mello AFM, Machado CMB, Neyra LCR, Ocán-Torres DY, Barros RN, Medeiros MC, Soccol CR, Vandenberghe LP de S. 2025. Biorefinery-based production of biodegradable bioplastics: advances and challenges in circular bioeconomy. npj Mater Sustain 3:42. doi:10.1038/s44296-025-00086-4

[4] Choi SY, Lee Y, Yu HE, Cho IJ, Kang M, Lee SY. 2023. Sustainable production and degradation of plastics using microbes. Nat Microbiol 8:2253–2276. doi:10.1038/s41564-023-01529-1 38030909

[5] Qin J, Wang X, Zheng Z, Ma C, Tang H, Xu P. 2010. Production of L-lactic acid by a thermophilic *Bacillus* mutant using sodium hydroxide as neutralizing agent. Bioresour Technol 101:7570–7576. doi:10.1016/j.biortech.2010.04.037 20488697

[6] Nakano S, Ugwu CU, Tokiwa Y. 2012. Efficient production of D-(-)-lactic acid from broken rice by *Lactobacillus delbrueckii* using Ca(OH)_2_ as a neutralizing agent. Bioresour Technol 104:791–794. doi:10.1016/j.biortech.2011.10.017 22093977

[7] Maas RHW, Bakker RR, Jansen MLA, Visser D, de Jong E, Eggink G, Weusthuis RA. 2008. Lactic acid production from lime-treated wheat straw by *Bacillus coagulans*: neutralization of acid by fed-batch addition of alkaline substrate. Appl Microbiol Biotechnol 78:751–758. doi:10.1007/s00253-008-1361-1 PMC2270352 18247027

[8] Yang P-B, Tian Y, Wang Q, Cong W. 2015. Effect of different types of calcium carbonate on the lactic acid fermentation performance of *Lactobacillus lactis*. Biochem Eng J 98:38–46. doi:10.1016/j.bej.2015.02.023

[9] Zhang B, Li R, Yu L, Wu C, Liu Z, Bai F, Yu B, Wang L. 2023. L-lactic acid production via sustainable neutralizer-free route by engineering acid-tolerant yeast *Pichia kudriavzevii*. J Agric Food Chem 71:11131–11140. doi:10.1021/acs.jafc.3c03163 37439413

[10] Tian X, Wang Y, Chu J, Zhuang Y, Zhang S. 2014. L-Lactic acid production benefits from reduction of environmental osmotic stress through neutralizing agent combination. Bioprocess Biosyst Eng 37:1917–1923. doi:10.1007/s00449-014-1166-9 24633312

[11] Yen H-W, Chen T-J, Pan W-C, Wu H-J. 2010. Effects of neutralizing agents on lactic acid production by *Rhizopus oryzae* using sweet potato starch. World J Microbiol Biotechnol 26:437–441. doi:10.1007/s11274-009-0186-0

[12] Huang X, Tian W, Wang X, Qin J. 2023. Time-resolved transcriptomic and proteomic profiling of *Heyndrickxia coagulans* during NaOH-buffered L-lactic acid production. Front Microbiol 14:1296692. doi:10.3389/fmicb.2023.1296692 PMC10716427 38094625

[13] Qin J, Wang X, Wang L, Zhu B, Zhang X, Yao Q, Xu P. 2015. Comparative transcriptome analysis reveals different molecular mechanisms of *Bacillus coagulans* 2-6 response to sodium lactate and calcium lactate during lactic acid production. PLoS One 10:e0124316. doi:10.1371/journal.pone.0124316 PMC4398400 25875592

[14] Liang S, Gao D, Liu H, Wang C, Wen J. 2018. Metabolomic and proteomic analysis of D-lactate-producing *Lactobacillus delbrueckii* under various fermentation conditions. J Ind Microbiol Biotechnol 45:681–696. doi:10.1007/s10295-018-2048-y 29808292

[15] Abdel-Rahman MA, Tashiro Y, Sonomoto K. 2013. Recent advances in lactic acid production by microbial fermentation processes. Biotechnol Adv 31:877–902. doi:10.1016/j.biotechadv.2013.04.002 23624242

[16] Lu Z, He F, Shi Y, Lu M, Yu L. 2010. Fermentative production of L(+)-lactic acid using hydrolyzed acorn starch, persimmon juice and wheat bran hydrolysate as nutrients. Bioresour Technol 101:3642–3648. doi:10.1016/j.biortech.2009.12.119 20116239

[17] Ma K, Maeda T, You H, Shirai Y. 2014. Open fermentative production of L-lactic acid with high optical purity by thermophilic *Bacillus coagulans* using excess sludge as nutrient. Bioresour Technol 151:28–35. doi:10.1016/j.biortech.2013.10.022 24201025

[18] Zhang C, Zhou C, Assavasirijinda N, Yu B, Wang L, Ma Y. 2017. Non-sterilized fermentation of high optically pure D-lactic acid by a genetically modified thermophilic *Bacillus coagulans* strain. Microb Cell Fact 16:213. doi:10.1186/s12934-017-0827-1 PMC5702109 29178877

[19] Liu Z, Chen M, Hu J, Wang Y, Chen Y. 2025. Minimization of proteome reallocation explains metabolic transition in hierarchical utilization of carbon sources. mSystems 10:e00690–25. doi:10.1128/msystems.00690-25 PMC12282068 40586598

[20] Tian W, Qin J, Lian C, Yao Q, Wang X. 2022. Identification of a major facilitator superfamily protein that is beneficial to L-lactic acid production by *Bacillus coagulans* at low pH. BMC Microbiol 22:310. doi:10.1186/s12866-022-02736-2 PMC9764580 36536285

[21] Cabrera MN, Vila E, Liguori A, D’Andrada C, Moure S, Guigou M, Cebreiros F, Rodao JM, Camesasca L, Ferrari MD, Lareo C. 2024. Purification of xylosaccharides from eucalyptus residues for l‐lactic acid production by *Weizmannia coagulans*. Biofuel Bioprod Bior 18:1902–1916. doi:10.1002/bbb.2662

[22] Yan Y, Shan W, Zhang C, Wu Y, Xing X, Chen J, Hu W. 2024. Strain engineering of *Bacillus coagulans* with high osmotic pressure tolerance for effective L-lactic acid production from sweet sorghum juice under unsterile conditions. Bioresour Technol 400:130648. doi:10.1016/j.biortech.2024.130648 38561153

[23] Liu Z, Wang Y. 2025. Systems biology analysis of the effect of pH on lactate fermentation in *Bacillus coagulans* under mixed carbon sources. Biotechnol Bioeng 122:2559–2573. doi:10.1002/bit.70001 40503922

[24] Saw CY, Chang TJ, Chen PY, Dai FJ, Lau YQ, Chen TY, Chau CF. 2019. Presence of *Bacillus coagulans* spores and vegetative cells in rat intestine and feces and their physiological effects. Biosci Biotechnol Biochem 83:2327–2333. doi:10.1080/09168451.2019.1651628 31403387

[25] Dooley D, Ryu S, Giannone RJ, Edwards J, Dien BS, Slininger PJ, Trinh CT. 2024. Expanded genome and proteome reallocation in a novel, robust *Bacillus coagulans* strain capable of utilizing pentose and hexose sugars. mSystems 9:e0095224. doi:10.1128/msystems.00952-24 PMC11575207 39377583

[26] Abdel-Rahman MA, Hassan S-D, Alrefaey HMA, Elsakhawy T. 2021. Efficient Co-utilization of biomass-derived mixed sugars for lactic acid production by *Bacillus coagulans* Azu-10. Fermentation 7:28. doi:10.3390/fermentation7010028

[27] Abdel-Rahman MA, Hassan S-D, Fouda A, Radwan AA, Barghoth MG, Desouky SG. 2021. Evaluating the effect of lignocellulose-derived microbial inhibitors on the growth and lactic acid production by *Bacillus coagulans* Azu-10. Fermentation 7:17. doi:10.3390/fermentation7010017

[28] Chen Y, Dong F, Wang Y. 2016. Systematic development and optimization of chemically defined medium supporting high cell density growth of *Bacillus coagulans*. Appl Microbiol Biotechnol 100:8121–8134. doi:10.1007/s00253-016-7644-z 27262567

[29] Ogata S, Hongo M. 2000. Lysis induced by sodium ion and its relation to lytic enzyme systems in *Clostridium saccharoperbutylacetonicum*. Microbiology 81:315–323. doi:10.1099/00221287-81-2-315 4836124

[30] Thomas KJ III, Rice CV. 2014. Revised model of calcium and magnesium binding to the bacterial cell wall. Biometals 27:1361–1370. doi:10.1007/s10534-014-9797-5 PMC4299761 25315444

[31] Thomas KJ III, Rice CV. 2015. Equilibrium binding behavior of magnesium to wall teichoic acid. Biochim Biophys Acta 1848:1981–1987. doi:10.1016/j.bbamem.2015.05.003 PMC4554814 25969394

[32] Ostrowska-Czubenko J. 2002. Competitive binding of Na^+^ and Ca^2+^ ions to teichoic acid analogues. Colloid Poly Sci 280:1015–1020. doi:10.1007/s00396-002-0724-x

[33] Chen Y, Sun Y, Liu Z, Dong F, Li Y, Wang Y. 2020. Genome‐scale modeling for *Bacillus coagulans* to understand the metabolic characteristics. Biotechnol Bioeng 117:3545–3558. doi:10.1002/bit.27488 32648961

[34] Grigaitis P, Teusink B. 2022. An excess of glycolytic enzymes under glucose-limited conditions may enable *Saccharomyces cerevisiae* to adapt to nutrient availability. FEBS Lett 596:3203–3210. doi:10.1002/1873-3468.14484 36008883

[35] Neves AR, Ramos A, Nunes MC, Kleerebezem M, Hugenholtz J, de Vos WM, Almeida J, Santos H. 1999. *In vivo* nuclear magnetic resonance studies of glycolytic kinetics in *Lactococcus lactis*. Biotechnol Bioeng 64:200–212. doi:10.1002/(sici)1097-0290(19990720)64:2<200::aid-bit9>3.0.co;2-k 10397856

[36] Fernández M, Zúñiga M. 2006. Amino acid catabolic pathways of lactic acid bacteria. Crit Rev Microbiol 32:155–183. doi:10.1080/10408410600880643 16893752

[37] Lane S, Turner TL, Jin YS. 2023. Glucose assimilation rate determines the partition of flux at pyruvate between lactic acid and ethanol in *Saccharomyces cerevisiae*. Biotechnol J 18:e2200535. doi:10.1002/biot.202200535 36723451

[38] Zhang K, Zhang Z, Guo X, Guo R, Zhu L, Qiu X, Yu X, Chai J, Gu C, Feng Z. 2023. Changes in nutrient consumption patterns of *Lactobacillus fermentum* mediated by sodium lactate. J Sci Food Agric 103:1775–1783. doi:10.1002/jsfa.12295 36305089

[39] Wu K, Liu H, Zhou Y, Sun M, Mao R, Jiang Y, Kerkhoven EJ, Chen Y, Nielsen J, Tang H, Li F. 2026. Systematically exploring yeast metabolism through retrobiosynthesis and deep learning. Nat Catal 9:434–447. doi:10.1038/s41929-026-01523-w

[40] Padan E, Venturi M, Gerchman Y, Dover N. 2001. Na^+^/H^+^ antiporters. Biochimica et Biophysica Acta (BBA) - Bioenergetics 1505:144–157. doi:10.1016/S0005-2728(00)00284-X 11248196

[41] Ballmoos C, Wiedenmann A, Dimroth P. 2009. Essentials for ATP Synthesis by F1F0 ATP Synthases. Annu Rev Biochem 78:649–672. doi:10.1146/annurev.biochem.78.081307.104803 19489730

[42] Boecker S, Slaviero G, Schramm T, Szymanski W, Steuer R, Link H, Klamt S. 2021. Deciphering the physiological response of *Escherichia coli* under high ATP demand. Mol Syst Biol 17:e10504. doi:10.15252/msb.202110504 PMC8686765 34928538

[43] Liu R, Zheng R, Liu G, Sun C. 2020. The cyclic lipopeptides suppress the motility of *Vibrio alginolyticus* via targeting the Na^+^ -driven flagellar motor component MotX. Environ Microbiol 22:4424–4437. doi:10.1111/1462-2920.15144 32608186

[44] Tierney AR, Rather PN. 2019. Roles of two-component regulatory systems in antibiotic resistance. Future Microbiol 14:533–552. doi:10.2217/fmb-2019-0002 PMC6526388 31066586

[45] Groisman EA. 2016. Feedback control of two-component regulatory systems. Annu Rev Microbiol 70:103–124. doi:10.1146/annurev-micro-102215-095331 PMC8380452 27607549

[46] Hoch JA. 2017. A life in *Bacillus subtilis* signal transduction. Annu Rev Microbiol 71:1–19. doi:10.1146/annurev-micro-030117-020355 28886686

[47] Luo YY, Yang JK, Zhu ML, Liu CJ, Li HY, Lu ZB, Pan WZ, Zhang ZH, Bi W, Zhang KQ. 2012. The group III two-component histidine kinase AlHK1 is involved in fungicides resistance, osmosensitivity, spore production and impacts negatively pathogenicity in *Alternaria longipes*. Curr Microbiol 64:449–456. doi:10.1007/s00284-012-0093-8 22349956

[48] Memmi G, Nair DR, Cheung A. 2012. Role of ArlRS in autolysis in methicillin-sensitive and methicillin-resistant *Staphylococcus aureus* strains. J Bacteriol 194:759–767. doi:10.1128/JB.06261-11 PMC3272946 22139508

[49] Fournier B, Hooper DC. 2000. A new two-component regulatory system involved in adhesion, autolysis, and extracellular proteolytic activity of *Staphylococcus aureus*. J Bacteriol 182:3955–3964. doi:10.1128/JB.182.14.3955-3964.2000 PMC94580 10869073

[50] Alodaini D, Hernandez-Rocamora V, Boelter G, Ma X, Alao MB, Doherty HM, Bryant JA, Moynihan P, Moradigaravand D, Glinkowska M, Vollmer W, Banzhaf M. 2024. Reduced peptidoglycan synthesis capacity impairs growth of *E. coli* at high salt concentration. mBio 15:e0032524. doi:10.1128/mbio.00325-24 PMC11005333 38426748

[51] Dufour D, Lévesque CM. 2013. Cell death of *Streptococcus* mutans induced by a quorum-sensing peptide occurs via a conserved streptococcal autolysin. J Bacteriol 195:105–114. doi:10.1128/JB.00926-12 PMC3536168 23104806

[52] Jan Nexson Parhusip A, Boing Sitanggang A. 2011. Antimicrobial activity of melinjo seed and peel extragainst selected pathogenic bacact (*Gnetum gnemon*) against selected pathogenic bacteria. MI 5:103–112. doi:10.5454/mi.5.3.2

[53] Wang Y, Hao F, Lu W, Suo X, Bellenger E, Fu N, Jeantet R, Chen XD. 2020. Enhanced thermal stability of lactic acid bacteria during spray drying by intracellular accumulation of calcium. J Food Eng 279:109975. doi:10.1016/j.jfoodeng.2020.109975

[54] Huang S, Chen XD. 2013. Significant effect of Ca^2+^ on improving the heat resistance of lactic acid bacteria. FEMS Microbiol Lett 344:31–38. doi:10.1111/1574-6968.12151 23617813

[55] Su Y, Zheng X, Zhao Q, Fu N, Xiong H, Wu WD, Chen XD. 2019. Spray drying of *Lactobacillus rhamnosus* GG with calcium-containing protectant for enhanced viability. Powder Technol 358:87–94. doi:10.1016/j.powtec.2018.09.082

[56] Wright CT, Klaenhammer TR. 1983. Survival of *Lactobacillus bulgaricus* during freezing and freeze‐drying after growth in the presence of calcium. J Food Sci 48:773–777. doi:10.1111/j.1365-2621.1983.tb14896.x

[57] Radoš D, Donati S, Lempp M, Rapp J, Link H. 2022. Homeostasis of the biosynthetic *E. coli* metabolome. iScience 25:104503. doi:10.1016/j.isci.2022.104503 PMC9218372 35754712

[58] Hui S, Silverman JM, Chen SS, Erickson DW, Basan M, Wang J, Hwa T, Williamson JR. 2015. Quantitative proteomic analysis reveals a simple strategy of global resource allocation in bacteria. Mol Syst Biol 11:784. doi:10.15252/msb.20145697 PMC4358657 25678603

[59] Spriggs KA, Bushell M, Willis AE. 2010. Translational regulation of gene expression during conditions of cell stress. Mol Cell 40:228–237. doi:10.1016/j.molcel.2010.09.028 20965418

[60] De Angelis M, Mariotti L, Rossi J, Servili M, Fox PF, Rollán G, Gobbetti M. 2002. Arginine catabolism by sourdough lactic acid bacteria: purification and characterization of the arginine deiminase pathway enzymes from *Lactobacillus sanfranciscensis* CB1. Appl Environ Microbiol 68:6193–6201. doi:10.1128/AEM.68.12.6193-6201.2002 PMC134416 12450844

[61] Balakrishnan R, de Silva RT, Hwa T, Cremer J. 2021. Suboptimal resource allocation in changing environments constrains response and growth in bacteria. Mol Syst Biol 17:e10597. doi:10.15252/msb.202110597 PMC8687047 34928547

[62] Zhang YP, Vera JM, Xie D, Serate J, Pohlmann E, Russell JD, Hebert AS, Coon JJ, Sato TK, Landick R. 2019. Multiomic fermentation using chemically defined synthetic hydrolyzates revealed multiple effects of lignocellulose-derived inhibitors on cell physiology and xylose utilization in *Zymomonas mobilis*. Front Microbiol 10:2596. doi:10.3389/fmicb.2019.02596 PMC6853872 31787963

[63] Bujdoš D, Popelářová B, Volke DC, Nikel PI, Sonnenschein N, Dvořák P. 2023. Engineering of *Pseudomonas putida* for accelerated co-utilization of glucose and cellobiose yields aerobic overproduction of pyruvate explained by an upgraded metabolic model. Metab Eng 75:29–46. doi:10.1016/j.ymben.2022.10.011 36343876

[64] Okano H, Hermsen R, Kochanowski K, Hwa T. 2020. Regulation underlying hierarchical and simultaneous utilization of carbon substrates by flux sensors in *Escherichia coli*. Nat Microbiol 5:206–215. doi:10.1038/s41564-019-0610-7 PMC6925339 31819215

[65] Lv X, Guo Y, Zhuang Y, Wang Y. 2015. Optimization and validation of an extraction method and HPAEC-PAD for determination of residual sugar composition in l -lactic acid industrial fermentation broth with a high salt content. Anal Methods 7:9076–9083. doi:10.1039/C5AY01703C

[66] Cipollina C, ten Pierick A, Canelas AB, Seifar RM, van Maris AJA, van Dam JC, Heijnen JJ. 2009. A comprehensive method for the quantification of the non-oxidative pentose phosphate pathway intermediates in *Saccharomyces cerevisiae* by GC–IDMS. Journal of Chromatography B 877:3231–3236. doi:10.1016/j.jchromb.2009.07.019 19647496

[67] Wang G, Chu J, Zhuang Y, van Gulik W, Noorman H. 2019. A dynamic model-based preparation of uniformly-^13^C-labeled internal standards facilitates quantitative metabolomics analysis of *Penicillium chrysogenum*. J Biotechnol 299:21–31. doi:10.1016/j.jbiotec.2019.04.021 31047964

[68] Liu X, Sun X, He W, Tian X, Zhuang Y, Chu J. 2019. Dynamic changes of metabolomics and expression of candicidin biosynthesis gene cluster caused by the presence of a pleiotropic regulator AdpA in *Streptomyces* ZYJ-6. Bioprocess Biosyst Eng 42:1353–1365. doi:10.1007/s00449-019-02135-4 31062087

[69] Seifar RM, Ras C, van Dam JC, van Gulik WM, Heijnen JJ, van Winden WA. 2009. Simultaneous quantification of free nucleotides in complex biological samples using ion pair reversed phase liquid chromatography isotope dilution tandem mass spectrometry. Anal Biochem 388:213–219. doi:10.1016/j.ab.2009.02.025 19250917

