# Supplementary material for: Sodium ion stress accelerates cell lysis in Bacillus coagulans by exacerbating energy deficit during carbon source transition
Source: Appl Environ Microbiol. 2026 Aug 5;92(9):e01314-26. doi: 10.1128/aem.01314-26 (PMC13599482; doi:10.1128/aem.01314-26)
Supplement: Supplementary file 1 [file aem.01314-26-s0001.docx]

*Supplementary Information for*:

**Sodium Ion Stress Accelerates Cell Lysis in** ***Bacillus coagulans* by Exacerbating Energy Deficit during Carbon Source Transition**

Zhihao Liu^1, 2, 3, 4^, Yingping Zhuang^1, 2, 3, 4^, Yonghong Wang^4#^, Guan Wang^1, 2, 3, 4#^

1 State Key Laboratory of Photoelectric Conversion and Utilization of Solar Energy, Qingdao Institute of Bioenergy and Bioprocess Technology, Chinese Academy of Sciences, Qingdao, 266101, China

2 Shandong Energy Institute, Qingdao, 266101, China

3 Qingdao New Energy Shandong Laboratory, Qingdao, 266101, China

4 State Key Laboratory of Bioreactor Engineering, East China University of Science and Technology, Shanghai 200237, China

\# Corresponding authors:

Yonghong Wang (yhwang@ecust.edu.cn)

Guan Wang (guanwang@ecust.edu.cn)

**Metabolite abbreviations:**

T6P, trehalose 6‐phosphate; G6P, glucose 6‐phosphate; F6P, fructose 6‐phosphate; FBP, fructose 1,6‐bisphosphate; DHAP, dihydroxyacetone phosphate; G3P, glyceraldehyde 3‐phosphate; 13BPG, glycerate 1,3‐bisphosphate; 3PG, 3-phosphoglycerate; 2PG, 2-Phospho-D-glycerate; PEP, phosphoenolpyruvate; 6PG, gluconate 6‐phosphate; E4P, erythrose 4‐phosphate; S7P, sedoheptulose 7-phosphate; R5P, ribose 5‐phosphate; RL5P, ribulose 5‐phosphate; XL5P, xylulose 5-phosphate; Acetyl-P, Acetyl phosphate.

**Fig. S1 Observation of cell lysis under different neutralizer conditions.** The 12 h and 24 h time points represent the P_lag_ and P_tre_ phases of the fermentation process, respectively.

**Fig. S2 Characteristics of mixed carbon source lactic acid fermentation of *Bacillus coagulans* under different fermentation conditions.** (a) Fermentation results of calcium hydroxide and sodium hydroxide mixture as neutralizer. (b) Fermentation results under the condition of complex medium and sodium hydroxide as neutralizer. The results are averaged from three biologically replicates.

**Fig. S3 Changes in the concentration of substances in medium under different neutralizer conditions.** (a) Changes in extracellular amino acid concentrations during fermentation. (b) Changes in calcium and sodium ion concentrations under calcium hydroxide and sodium hydroxide conditions derived from the amount of added alkali. The results are averaged from three biologically replicates.

**Fig. S4 Transcriptome analysis of the different neutralizer conditions.** (a) Principal component analysis (PCA) plotting of transcriptomic data for three phases. The three points of the same color represent three biological repetitions of the sampling point. (b) Correlation analysis. Samples were analyzed for pairwise correlation using Pearson's correlation coefficient. The transcriptome samples were collected at 7 h, 12 h, and 48 h, corresponding to P_gluc_, P_lag_, and P_tre_, respectively.

**Fig. S5 Transcriptomic comparison of purine metabolism in P_lag_ between different neutralizer conditions.** Green boxes indicate genes significantly downregulated in sodium hydroxide compared to calcium hydroxide, while red boxes denote significantly upregulated genes.

**Fig. S6 Intracellular ATP/ADP concentration ratios under two different neutralizer conditions.** Statistical analysis between the two neutralizers was performed using two-sided t-test. Statistically significant differences are described as follows: *** FDR <0.001, * FDR <0.05, ns is no significant.

**Fig. S7 Dynamic differences in gene expression during fermentation process under different neutralizers.** The colored square plot in the lower right corner of the figure represents significant (q‑value < 0.05) gene expression trends during the fermentation process, while the bubble plot illustrates the KEGG enrichment analysis results of the gene clusters corresponding to these significant trends. The left panel presents the results under calcium hydroxide conditions, while the right panel displays those under sodium hydroxide conditions.

**Fig. S8 Characteristics of mixed carbon source lactic acid fermentation of** **evolved *Bacillus coagulans* under calcium hydroxide conditions.** The results are averaged from three biologically replicates.
